# Molecular genetic characterization of cblC defects in 126 pedigrees and prenatal genetic diagnosis of pedigrees with combined methylmalonic aciduria and homocystinuria

**DOI:** 10.1186/s12881-018-0666-x

**Published:** 2018-08-29

**Authors:** Shuang Hu, Shiyue Mei, Ning Liu, Xiangdong Kong

**Affiliations:** The Center for Genetics and Prenatal Diagnosis, The First Affiliated Hospital of Zhengzhou University, Jianshe Road, Zhengzhou, 450052 China

**Keywords:** Combined methylmalonic aciduria and homocystinuria, *MMACHC*, cblC, Variant, Prenatal genetic diagnosis

## Abstract

**Background:**

We sought to analyse *MMACHC* variants among 126 pedigrees with cobalamin (cbl) C deficiency and combined methylmalonic aciduria and homocystinuria by Sanger sequencing, characterize the spectrum of *MMACHC* gene variants, and perform prenatal genetic diagnosis by chorionic villus sampling among these pedigrees.

**Methods:**

Peripheral blood was collected from 126 probands and their parents who visited the Genetic Counseling Clinic at our hospital between January 2014 and December 2017, and DNA was extracted from the blood. Then, we amplified the coding sequence and splicing regions of the *MMACHC* gene by PCR, and the PCR products were further sequenced to detect the variants in each pedigree. In 62 families, pregnant women were subjected to chorionic villus sampling for prenatal genetic diagnosis.

**Results:**

In total, 31 distinct variants were detected in the 126 pedigrees, and the most frequent variants were c.609G > A (p.Trp203Ter), c.658_660delAAG (p.Lys220del), c.567dupT (p.Ile190Tyrfs*13) and c.80A > G (p.Gln27Arg). Two of these variants have not been previously reported in the literature. One variant [c.463_465delGGG (p.Gly155del)] is a small-scale deletion, and the other variant [c.637G>T(p.Glu213Ter)] is a nonsense mutation. Among the 62 pedigrees who received a prenatal diagnosis, 16 foetuses were normal, 34 foetuses were carriers of heterozygous variants, and the remaining 12 foetuses harboured compound heterozygous variants or homozygous variants. Couples whose foetuses were normal or carriers continued the pregnancy, whereas couples whose foetuses harboured compound heterozygous variants or homozygous variants decided to terminate the pregnancy. The follow-up results were consistent with the prenatal diagnosis.

**Conclusions:**

Two novel *MMACHC* variants were identified, and prenatal genetic diagnosis is an accurate and convenient method that helps avoid the delivery of combined methylmalonic aciduria and homocystinuria patients.

**Electronic supplementary material:**

The online version of this article (10.1186/s12881-018-0666-x) contains supplementary material, which is available to authorized users.

## Background

Methylmalonic acidaemia or aciduria (MMA), which is the most common inborn error of organic acid metabolism [1], is inherited as an autosomal recessive disease caused by deficiency in methylmalonyl coenzyme A mutase (MCM) or intracellular cobalamin (cbl) metabolism [2]. Based on the patients’ biochemical features, MMA can be classified as isolated MMA or combined methylmalonic aciduria and homocystinuria, and the latter consists of five subtypes, including cblC, cblD, cblF, cblJ and cblX deficiencies [3, 4]. These defects result in methylcobalamin and adenosylcobalamin dysfunction, leading to the subsequent accumulation of methylmalonic acid and homocysteine in the blood and urine, which is a biochemical hallmark of this disorder [5, 6]. In China, the most commonly affected patients have combined methylmalonic acidaemia and homocystinuria, accounting for approximately 80% of all MMA cases. Among the subtypes, the cblC defect (MIM 277400) is the most common [7] and is caused by mutations in the *MMACHC* gene, which is located in chromosome region 1p34.1; the gene contains five exons and encodes a 282 amino acid protein [8].

An increasing number of cblC patients have benefited from an early diagnosis and better outcomes through expanded newborn screening, particularly gas chromatography-mass spectrometry (GC-MS) [9]. Despite the reduced mortality and morbidity in MMA children, their quality of life remains very low, and the families of these patients suffer a significant economic burden. The most effective measure to reduce the social and family pressure is to avoid the birth of children with MMA by prenatal diagnosis. Measuring the activity of methylmalonyl CoA mutase in amniotic fluid, amniotic fluid cells and chorionic cells and quantifying cbl metabolites in amniotic fluid cells have been employed in prenatal diagnosis [10, 11]. Combined with these biochemical analyses, genetic analyses are also currently used. Other studies report that prenatal diagnosis is performed through simple genetic diagnosis. Here, our study employs a simple genetic diagnosis.

To enhance our knowledge of this disorder, we recruited 126 pedigrees with combined methylmalonic acidaemia and homocystinuria who visited the Genetic Counseling Clinic of the First Affiliated Hospital of Zhengzhou University between January 2014 and December 2017. The *MMACHC* variants in the probands and their parents were analysed. Among these pedigrees, 62 pregnant mothers underwent prenatal genetic diagnosis. The primary aim of this study was to provide a foundation for rapid and efficient genetic-based diagnosis, genetic counselling for patients with cblC deficiency, and prenatal diagnosis of MMA in China.

This study was approved by the Scientific and Experimental Research Medical Ethics Committee of the First Affiliated Hospital of Zhengzhou University. All analysed samples were obtained after signed informed consent was provided.

## Methods

### Pedigrees and MMA diagnosis

Our study includes samples from 126 couples who had a child birth history of MMA or children with MMA whose diagnosis was established based on the clinical presentation, positive methylmalonic acid results or newborn screening by GC-MS (GC-MS, QP2010, Schimadzu, Japan). The patients were recruited from the Genetic Counseling Clinic of the First Affiliated Hospital of Zhengzhou University between January 2014 and December 2017. No patients were the product of consanguineous marriages.

### DNA extraction

Peripheral blood samples were collected from 90 probands and 126 couples. For families with a deceased-proband, samples were only collected from the parents. Chorionic villus sampling from pregnant women was performed with ultrasonic guidance at 11 to 14 weeks of gestation. Genomic DNA was extracted from the peripheral blood samples and chorionic villus samples using a DNA extraction kit (Omega blood/tissue DNA kit, Georgia, United States) according to the manufacturer’s instructions.

### PCR and sanger sequencing

The polymerase chain reaction (PCR) primers were designed according to previously published data [12]. The coding exons and splicing regions of the *MMACHC* gene were amplified by PCR, and the PCR products were subsequently sequenced bi-directionally using an ABI 3130-xl gene analyser (Life Technologies, Carlsbad, CA, United States). To identify the nucleotides, the sequences were aligned and inspected using a reference sequence from Ensemble (NM_015506) (http://asia.ensembl.org/).

### Genotype analysis

By searching the HGMD database (http://www.hgmd.cf.ac.uk/ac/index.php) and SNP database (http://www.ncbi.nlm.nih.gov/), the novel variants were named according to the international gene mutation nomenclature system (http://www.HGVS.org/varnomen). Mutation taster, Polymorphism phenotyping (PolyPhen) and PROVEAN were calculated to predict the pathogenic effects of the variations of interest.

### Prenatal diagnosis

After ascertaining the genotype of the probands and their parents, prenatal diagnosis was performed by chorionic villus sampling of pregnant women. To exclude contamination from the mother, a PowerPlex 16 HS System kit (Promega, Madison, WI, USA) was used. The results were analysed using ABI 3130xl and GeneMapper v3.2 software.

### Follow-up

Umbilical cord blood was collected for genetic diagnosis of the foetuses.

## Results

### *MMACHC* gene variant spectrum

The 4 coding exons of the *MMACHC* gene and their flanking regions in each sample were analysed. Of the fully genotyped patients, 94 cases harboured compound heterozygous variants, and the remaining 32 cases harboured homozygous variants. All parents were heterozygous carriers.

In total, we identified 31 different variants among the 126 patients recruited for our study. Two of these variants were novel, whereas the remaining 29 variants have been previously described. A complete list of all variants is provided in Table 1. The two allele variants of 126 pedigrees in our study is provided in (Additional file 1: Table S3). Among these variants, the most prevalent variant was c.609G > A(p.Trp203Ter) (48.41%), followed by c.658_660delAAG(p.Lys220del) (13.49%), c.567dupT(p.Ile190Tyrfs*13) (6.75%), c.80A > G (p.Gln27Arg) (5.95%), c.482G > A(p.Arg161Gln) (5.95%) and c.217C > T(p.Arg73Ter) (3.97%). None of the variants described above were identified among 100 healthy control subjects.

**Table 1:** *MMACHC* gene variants for 126 pedigrees

No.	cDNA change	Amino acid change	Exon	Effect	Frequency	Percentage
1	c.1A > G	p.Met1Val	1	missense variant	1	0.40%
2	c.80A > G	p.Gln27Arg	1	missense variant	15	5.95%
3	c.81 + 1G > A	splicing region	intron1	splicing variant	1	0.40%
4	c.217C > T	p.Arg73Ter	2	nonsense variant	10	3.97%
5	c.271dupA	p.Arg91Lysfs*14	2	frameshift variant	2	0.79%
6	c.315C > G	p.Tyr105Ter	3	nonsense variant	1	0.40%
7	c.328_331delAACC	p.Asn110Aspfs*13	3	frameshift variant	1	0.40%
8	c.331C > T	p.Arg111Ter	3	nonsense variant	2	0.79%
9	c.365A > T	p.His121Leu	3	missense variant	1	0.40%
10	c.394C > T	p.Arg132Ter	3	nonsense variant	6	2.38%
11	c.440_441delGT	p.Cys149Hisfs*32	4	frameshift variant	1	0.40%
12	c.445_446delTG	p.Cys149Hisfs*32	4	frameshift variant	5	1.98%
13	c.445_446insA	p.Cys149Ter	4	frameshift variant	2	0.79%
14	c.445_446delT	p.Cys149Alafs*15	4	frameshift variant	1	0.40%
15	c.463_465delGGG	p.Gly155del	4	deletion variant	1	0.40%
16	c.463G > C	p.G155R	4	missense variant	1	0.40%
17	c.465_467delGGG	p.Gly155del	4	deletion variant	1	0.40%
18	c.467G > A	p.Gly156Ala	4	missense variant	2	0.79%
19	c.481C > T	p.Arg161Ter	4	nonsense variant	5	1.98%
20	c.482G > A	p.Arg161Gln	4	missense variant	11	4.37%
21	c.565C > T	p.Arg189Cys	4	missense variant	1	0.40%
22	c.567dupT	p.Ile190Tyrfs*13	4	frameshift variant	17	6.75%
23	c.599G > A	p.Trp200Ter	4	nonsense variant	1	0.40%
24	c.609G > A	p.Trp203Ter	4	nonsense variant	122	48.41%
25	c.615C > A	p.Tyr205Ter	4	nonsense variant	1	0.40%
26	c.617G > A	p.Arg206Gln	4	missense variant	1	0.40%
27	c.626dupT	p.Thr210Aspfs*35	4	frameshift variant	1	0.40%
28	c.637G>T	p.Glu213Ter	4	nonsense variant	2	0.79%
29	c.658_660delAAG	p.Lys220del	4	deletion variant	34	13.49%
30	c.666C > A	p.Tyr222Ter	4	nonsense variant	1	0.40%
31	c.683C > T	p.Ala228Val	4	missense variant	1	0.40%

The variants can be classified into the following different types: missense variants, nonsense variants, splicing variants, small insertions, and small deletions. The spectrum of these distinct variants was distributed throughout the 4 coding exons and splicing regions of the *MMACHC* gene. Many variants were located in exon 4 (84.13%), and variants occurring in other exons were less frequent.

Among the 31 different variants, two variants, including the nonsense variant c.637G>T resulting in p.Glu213Ter and the small deletion variant c.463_465delGGG resulting in p.Gly155del, were novel variants. These two variants have not been reported in the HGMD database (http://www.hgmd.org) or the SNP database (http://www.ncbi.nlm.nih.gov/SNP). Moreover, according to the prediction tools PolyPhen and Mutation taster, all novel variants were predicted to be disease causing. Regarding the c.463_465delGGG variant, a conservation analysis of the amino acid sequence of *MMACHC* from humans and other species, such as *P. troglodytes*, *M. mulatta*, *F. catus*, and *M. musculus*, revealed that the G155 residue is highly conserved. The deletion of this amino acid may cause the loss of original protein features. The c.637G>T variant is a nonsense variant leading to the translation of a significantly truncated protein. The above-mentioned analysis further confirmed the pathogenicity of these mutations.

### Prenatal diagnosis and follow-up

After ascertaining the genotype of these pedigrees, prenatal diagnosis was performed by chorionic villus sampling of pregnant women. Contamination from the mother was prevented using a PowerPlex 16 HS System kit (Promega, Madison, WI, USA).

Among the 62 pedigrees who received prenatal diagnosis, 16 foetuses were normal, 34 foetuses were carriers of heterozygous variants, and the remaining 12 foetuses harboured compound heterozygous variants or homozygous variants (Table 2). Couples whose foetuses were normal or carriers continued the pregnancy, whereas couples whose foetuses harboured compound heterozygous variants or homozygous variants decided to terminate the pregnancy. The gene analysis of the foetuses’ umbilical cord blood was consistent with the prenatal diagnosis. In addition, no abnormal results were noted in newborns screened by GC-MS.

**Table 2:** The genotypes of 62 pedigrees who have performed the prenatal genetic diagnosis

No.	Proband	Mutation maternal	Mutation paternal	Futus genotype
1	Q27R/R161Q	p.Q27R	p.R161Q	p.Q27R/−
2	p.R73X/p.Lys220del	p.R73X	p.Lys220del	p.R73X/−
3	c.463_465delGGG/p.W203X	c.463_465delGGG	p.W203X	−/−
4	p.W203X/p.G156D	p.W203X	p.G156D	p.W203X/p.G156D
5	c.626dupT/p.W203X	c.626dupT	p.W203X	c.626dupT /−
6	p.Lys220del/p.W203X	p.Lys220del	p.W203X	p.Lys220del/p.W203X
7	p.W203X/ c.445_446delTG	p.W203X	c.445_446delTG	p.W203X/ c.445_446delTG
8	p.R132X/p.W203X	p.R132X	p.W203X	p.R132X/−
9	p.W203X/p.E213X	p.W203X	p.E213X	p.W203X/−
10	p.K220del/p.K220del	p.K220del	p.K220del	p.K220del/p.K220del
11	p.W203X/p.W203X	p.W203X	p.W203X	p.W203X/−
12	p.W203X/p.R161X	p.W203X	p.R161X	−/−
13	p.W203X/p.W203X	p.W203X	p.W203X	p.W203X/−
14	p.K220del/c.567dupT	p.K220del	c.567dupT	p.K220del/−
15	p.R132X/p.Y205X	p.R132X	p.Y205X	p.R132X/p.Y205X
16	p.R73X /p.R73X	p.R73X	p.R73X	p.R73X/−
17	c.445_446insA/c.567dupT	c.445_446insA	c.567dupT	c.567dupT/−
18	p.R73X/p.W203X	p.R73X	p.W203X	p.W203X/−
19	c.567dupT/p.W203X	c.567dupT	p.W203X	c.567dupT/p.W203X
20	p.W203X/p.K220del	p.W203X	p.K220del	p.W203X/−
21	p.G156A/p.W203X	p.G156A	p.W203X	p.G156A/−
22	p.W203X/p.W203X	p.W203X	p.W203X	p.W203X/−
23	p.W203X/p.R161X	p.W203X	p.R161X	p.W203X/−
24	c.567dupT/p.R132X	c.567dupT	p.R132X	p.R132X/−
25	p.W203X/p.W203X	p.W203X	p.W203X	p.W203X/−
26	c.440_441delGT/p.W203X	c.440_441delGT	p.W203X	p.W203X/−
27	p.W203X/p.K220del	p.W203X	p.K220del	p.W203X/p.K220del
28	p.Q27R/p.K220del	p.Q27R	p.K220del	p.Q27R/p.K220del
29	c.567dupT/p.W203X	c.567dupT	p.W203X	p.W203X/−
30	p.Q27R/p.Q27R	p.Q27R	p.Q27R	p.Q27R/p.Q27R
31	c.567dupT/p.K220del	c.567dupT	p.K220del	−/−
32	p.Y222X/p.Q27R	p.Y222X	p.Q27R	p.Y222X/−
33	p.W203X/p.K220del	p.W203X	p.K220del	−/−
34	p.K220del/p.W203X	p.K220del	p.W203X	−/−
35	p.Q27R/p.W203X	p.Q27R	p.W203X	−/−
36	p.W203X/p.K220del	p.W203X	p.K220del	p.K220del/−
37	p.W203X/p.R161Q	p.W203X	p.R161Q	p.R161Q/p.W203X
38	p.W203X/p.W203X	p.W203X	p.W203X	p.W203X/p.W203X
39	p.W203X/p.K220del	p.W203X	p.K220del	p.K220del/−
40	c.445_446insA/p.W203X	c.445_446insA	p.W203X	c.445_446insA/p.W203X
41	p.W203X/p.W203X	p.W203X	p.W203X	p.W203X/−
42	p.Q27R/c.567dupT	p.Q27R	c.567dupT	c.566_567insT/p.Q27R
43	p.W203X/p.W203X	p.W203X	p.W203X	p.W203X/−
44	p.Q27R/p.W203X	p.Q27R	p.W203X	p.W203X/−
45	p.R73X/p.W203X	p.R73X	p.W203X	−/−
46	p.R73X/p.W203X	p.R73X	p.W203X	−/−
47	p.K220del/p.W203X	p.K220del	p.W203X	p.W203X/−
48	p.W203X/p.W203X	p.W203X	p.W203X	p.W203X/−
49	p.K220del/p.W203X	p.K220del	p.W203X	p.W203X/−
50	p.Q27R/p.W203X	p.Q27R	p.W203X	p.Q27R/p.W203X
51	p.W203X/c.567dupT	p.W203X	c.567dupT	−/−
52	p.K220del/p.H121L	p.K220del	p.H121L	p.K220del/−
53	c.445-446delTG/p.W203X	c.445-446delTG	p.W203X	p.W203X/−
54	c.567dupT/p.K220del	c.567dupT	p.K220del	−/−
55	c.567dupT/p.W203X	c.567dupT	p.W203X	p.W203X/−
56	c.445-446delTG/p.W203X	c.445-446delTG	p.W203X	c.445-446delTG/p.W203X
57	p.Q27R/p.W203X	p.Q27R	p.W203X	p.Q27R/−
58	p.A228V/p.W203X	p.A228V	p.W203X	−/−
59	p.K220del/c.445-446delTG	p.K220del	c.445-446delTG	c.445-446delTG/−
60	p.W203X/p.W203X	p.W203X	p.W203X	−/−
61	p.Trp200X/p.W203X	p.Trp200X	p.W203X	p.W203X/−
62	p.W203X/p.R206Q	p.W203X	p.R206Q	p.W203X/p.R206Q

## Discussion

This study illustrates the spectrum of variants in the *MMACHC* gene in 126 pedigrees with cblC defects, including 62 families that choose to undergo prenatal diagnosis by chorionic villus sampling. In total, we identified 31 different variants in these 126 patients. Two of these variants were novel, whereas the remaining 29 variants have been previously reported in the literature.

MMA is the most prevalent organic aciduria involving intermediate metabolism due to enzymatic block. Based on the age at the presentation of the initial symptoms and diagnosis (from newborn to older than 20 years), patients are divided into the following two categories: early-onset and late-onset cblC deficiency. The latter is a rare disorder, and diagnosis is often delayed. Most reported patients with the cblC type present in the first year and typically in the neonatal period with lethargy, vomiting, hypotonia, respiratory distress and developmental delay, which is referred to as early-onset cblC [13, 14]. The other type is the late-onset cblC form, and patients with cblC exhibit ataxia, dementia, psychosis and other neurologic abnormalities generally after 4 years of age [15, 16].

Currently, newborns are screened by MS and GC-MS, and abnormalities in early-onset and late-onset children can be easily observed in urine and blood. Early diagnosis and treatment are beneficial in children with cblC defects, resulting in improved quality of life and reduced suffering from pain.

Treatment options like betaine, hydroxocobalamin, methylfolate, vitamin B6 and L-carnitine are applied to these patients clinically, the combined treatment can effectively improve the clinical manifestations in acute phase and alleviate biochemical abnormalities in hematuria. The median levels of serum homocysteine, and urine methylmalonic acid were significantly decreased (*p* < 0.01), from 97.3 μmol/L (ranged from 25.1 to 250 μmol/L) and 168.55 (ranged from 3.66 to 1032.82) before treatment to 43.8 μmol/L (ranged from 17 to 97.8 μmol/L) and 6.81 (ranged from 0 to 95.43) after treatment, respectively [17]. For those from a rural area they suffer more pain due to undertreatment, or late initiation of optimal treatment in China. Even with enough combined treatment, along with much lower levels of serum homocysteine and urine methylmalonic acid than before, their long-term outcomes are not encouraging, along with severe neurological sequelae in most patients.

To date, more than 90 mutations in this gene, including missense mutations, nonsense mutations, small indels, and splicing region mutations, have been reported in the literature [18]*.* Given the high allelic heterogeneity in MMA, the spectrum of variants differs in various populations worldwide. Thus, mutational analysis of MMA patients is crucial in different populations. Specifically, c.271dupA (21%), c.394C > T (21%) and c.609G > A (11%) are the most frequent variants in Europe based on a study conducted in 2014 [19]. Other studies have reported that the c.394C > T variant is the most common allele in Native American and Middle Eastern MMA patients [20, 21]. However, numerous children with the cblC type have been diagnosed as homozygous for the c.609G > A variant, and more importantly, these children were East Asians [12, 20]. Another Chinese study reported that 55.4% (51/92) of all variants were c.609G > A [22]. Similarly, in this study, c.609G > A accounted for 48.41% of the variants. Among the 32 homozygous variants, 29 variants were homozygous for c.609G > A. Thus, we further confirm that c.609G > A is a very common mutation in China. Following the most common c.609G > A variant is c.658_660delAAG, which accounted for 13.49% in this study and is also a very common mutation in Chinese patients [8].

Of the 31 different variants identified in this study, 2 variants were novel. One variant, i.e., the nonsense variant c.637G>T (p.Glu213Ter), results in a truncated protein at residue 213. The other variant, i.e., small deletion c.463_465delGGG (p.Gly155del), causes the deletion of a glycine at residue 155. The c.463G > C (Gly155Arg) and c.464G > A (Gly155Glu) variants have been previously reported to be pathogenic [23]. In addition, the highly conserved results of the PROVEAN and PolyPhen analyses of glycine 155 support the pathogenicity of the deletion. However, further research should be performed to verify our hypothesis.

Although early detection and treatment can improve the outcome in cblC patients, most probands die at a young age. Thus, preimplantation genetic diagnosis or screening (PGD/PGS) or prenatal diagnosis are the best currently available prevention methods. MMA can be diagnosed as early as the first trimester by chronic villus sampling, which is beneficial for families with probands.

However, undeniably, this study also has some limitations in terms of prenatal diagnosis. First, in families with deceased probands, we first detected the *MMACHC* gene variants in the parents. If both parents harboured a heterozygous variant of the *MMACHC* gene, we deduced that the proband genotype harboured compound heterozygous or homozygous variants. Direct testing of the probands was not performed. Second, traditional methods used for prenatal diagnosis include biochemical detections, such as measuring the activity of methylmalonyl CoA mutase in the amniotic fluid, amniotic fluid cells and chorionic cells and quantifying cbl metabolites in amniotic fluid cells. Our study exclusively performed a simple genetic diagnosis to these pedigrees with explicit homozygous or compound heterozygous mutations without traditional biochemical measurements. However, for those pedigrees with only one or no definite deleterious alleles, or some variants of unknown significance, we suggest them to other Hospital to perform prenatal diagnosis with biochemical testing, so here we do not collect them into our study, and we are unable to help them. Finally, pregnant women bearing MMA foetuses typically choose to terminate the pregnancy, which also represents a type of injury to the pregnant women. Overall, preimplantation genetic diagnosis can be useful for avoiding miscarriages or the induction of labour in early pregnancy and is a future direction in the development of MMA prenatal diagnosis.

## Conclusion

Two novel variants of the *MMACHC* gene were identified, and prenatal genetic diagnosis is an accurate and convenient method to help avoid the delivery of combined methylmalonic aciduria and homocystinuria patients.

## Additional file

Additional file 1: **Table S3**. The two allele variants of 126 pedigrees in our study. (DOCX 23 kb)

## Abbreviations

cblC: Cobalamin(cbl) C
GC-MS: Gas chromatography-mass spectrometry
MCM: Methylmalonyl coenzyme A mutase
MMA: Methylmalonic acidemia or aciduria
PCR: Polymerase chain reaction
PGD/PGS: Preimplantation genetic diagnosis or screening
