# Supplementary material for: Molecular genetic characterization of cblC defects in 126 pedigrees and prenatal genetic diagnosis of pedigrees with combined methylmalonic aciduria and homocystinuria
Source: BMC Med Genet. 2018 Aug 29;19:154. doi: 10.1186/s12881-018-0666-x (PMC6116561; doi:10.1186/s12881-018-0666-x)
Supplement: Supplementary file 1 — Table S3. The two allele variants of 126 pedigrees in our study. (DOCX 23 kb) [file 12881_2018_666_MOESM1_ESM.docx]

Additional file 1

**Table S3 The two allele variants of 126 pedigrees in our study.**

| No of pedigrees | Allele 1 | Allele 2 |
| --- | --- | --- |
| 1 | c.609G＞A | c.609G>A |
| 2 | c.609G>A | c.609G>A |
| 3 | c.217C>T | c.658_660delAAG |
| 4 | c.80A>G | c.658_660delAAG |
| 5 | c.658_660delAAG | c.609G>A |
| 6 | c.609G>A | c.609G>A |
| 7 | c.567_568 ins T | c.609G>A |
| 8 | c.609G>A | c.567_568insT |
| 9 | c.609G>A | c.609G>A |
| 10 | c.463_465delGGG | c.609G>A |
| 11 | c.315C>G | c.482G>A |
| 12 | c.467G>A | c.609G>A |
| 13 | c.609G>A | c.658_660delAAG |
| 14 | c.626dupT | c.609G>A |
| 15 | c.465_467delGGG | c.482G>A |
| 16 | c.609G>A | c.609G>A |
| 17 | c.658_660 delAAG | c.609G>A |
| 18 | c.609G>A | c.609G>A |
| 19 | c.609G>A | c.609G>A |
| 20 | c.445_446delTG | c.609G>A |
| 21 | c.394C>T | c.609G>A |
| 22 | c.463G>C | c.567dupT |
| 23 | c.658_660delAAG | c.658_660delAAG |
| 24 | c.609G>A | c.609G>A |
| 25 | c.609G>A | c.567dupT |
| 26 | c.609G>A | c.481C>T |
| 27 | c.609G>A | c.609G>A |
| 28 | c.658_660delAAG | c.567dupT |
| 29 | c.609G>A | c.609G>A |
| 30 | c.482G>A | c.567dupT |
| 31 | c.394C>T | c.615C>A |
| 32 | c.217C>T | c.217C>T R73X |
| 33 | c.445_446insA | c.567dupT |
| 34 | c.217C>T | c.609G>A |
| 35 | c.567dupT | c.609G>A |
| 36 | c.609G>A | c.656_658delAAG |
| 37 | c.609G>A | c.467G>A |
| 38 | c.482G＞A | c.609G＞A |
| 39 | c.609G>A | c.609G>A |
| 40 | c.565C>T | c.658_660delAAG |
| 41 | c.609G>A | c.609G>A |
| 42 | c.394C>T | c.445_446delTG |
| 43 | c.1A>G | c.609G>A |
| 44 | c.270_271insA | c.609G>A |
| 45 | c.481C>T | c.609G>A |
| 46 | c.567dupT | c.394C>T |
| 47 | c.658_660 delAAG | c.394C>T |
| 48 | c.609G>A | c.609G>A |
| 49 | c.482G>A | c.609G>A |
| 50 | c.440_441delGT | c.609G>A |
| 51 | c.331C>T | c.609G>A |
| 52 | c.609G>A | c.658_660delAAG |
| 53 | c.658-660delAAG | c.609G>A |
| 54 | c.80A>G | c.658_660delAAG |
| 55 | c.394C>T | c.658_660delAAG |
| 56 | c.328_331delAACC | c.609G>A |
| 57 | c.271dupA | c.658_660delAAG |
| 58 | c.567dupT | c.609G>A |
| 59 | c.80A>G | c.80A>G |
| 60 | c.567dupT | c.658_660delAAG |
| 61 | c.609G>A | c.609G>A |
| 62 | c.666C>A | c.80A>G |
| 63 | c.481C>T | c.609G>A |
| 64 | c.609G>A | c.658_660delAAG |
| 65 | c.609G>A | c.656-658del |
| 66 | c.609G>A | c.609G>A |
| 67 | c.658_660delAAG | c.609G>A |
| 68 | c.80A>G | c.609G>A |
| 69 | c.609G>A | c.658_660delAAG |
| 70 | c.609G>A | c.609G>A |
| 71 | c.482G>A | c.609G>A |
| 72 | c.217C>T | c.217C>T |
| 73 | c.609G>A | c.609G>A |
| 74 | c.609G>A | c.658_660delAAG |
| 75 | c.609G>A | c.445_446insA |
| 76 | c.609G>A | c.609G>A |
| 77 | c.566insT | c.80A>G/- |
| 78 | c.609G>A | c.609G>A |
| 79 | c.80A>G | c.609G>A |
| 80 | c.217C>T | c.609G>A |
| 81 | c.609G>A | c.609G>A |
| 82 | c.217C>T | c.609G>A |
| 83 | c.658_660delAAG | c.609G>A |
| 84 | c.609G>A | c.609G>A |
| 85 | c.658_660delAAG | c.609G>A |
| 86 | c.80A>G | c.609G>A |
| 87 | c.658_660delAAG | c.609G>A |
| 88 | c.609G>A | c.217C>T |
| 89 | c.609G>A | c.567dupT |
| 90 | c.80A＞G | c.658_660delAAG |
| 91 | c.658_660delAAG | c.365A＞T |
| 92 | c.80A＞G | c.658_660delAAG |
| 93 | c.445-446delTG | c.609G＞A |
| 94 | c.609G＞A | c.637G＞T |
| 95 | c.567_568insT | c.658_660delAAG |
| 96 | c.567_568insT | c.609G＞A |
| 97 | c.481C>T | c.609G＞A |
| 98 | c.482G>A | c.567_568insT |
| 99 | c.445_446delTG | c.609G＞A |
| 100 | c.80A＞G | c.609G＞A |
| 101 | c.567_568insT | c.658_660delAAG |
| 102 | c.609G＞A | c.609G＞A |
| 103 | c.609G＞A | c.609G＞A |
| 104 | c.609G＞A | c.609G＞A |
| 105 | c.81+1G＞A | c.609G＞A |
| 106 | c.482G>A | c.482G>A |
| 107 | c.445_446delT | c.609G>A |
| 108 | c.80A＞G | c.331C＞T |
| 109 | c.482G＞A | c.609G>A |
| 110 | c.609G>A | c.609G>A |
| 111 | c.609G>A | c.609G>A |
| 112 | c.609G>A | c.609G>A |
| 113 | c.481C>T | c.609G>A |
| 114 | c.217C>T | c.609G＞A |
| 115 | c.683C>T | c.609G>A |
| 116 | c.609G>A | c.609G>A |
| 117 | c.658_660delAAG | c.445_446delTG |
| 118 | c.658_660delAAG | c.609G>A |
| 119 | c.609G>A | c.609G>A |
| 120 | c.599G>A | c.609G>A |
| 121 | c.609G>A | c.658_660delAAG |
| 122 | c.609G>A | c.617G>A |
| 123 | c.80A>G | c.482G>A |
| 124 | c.609G>A | c.637G＞T |
| 125 | c.658_660delAAG | c.609G>A |
| 126 | c.80A>G | c.609G>A |
